# Reliability-oriented performance evaluation of PV inverters using wide bandgap semiconductors

**DOI:** 10.1371/journal.pone.0346925

**Published:** 2026-04-30

**Authors:** Sainadh Singh Kshatri, Javed Dhillon, Sachin Mishra, Naveen Kumar Sharma, Vinay Kumar Jadoun

**Affiliations:** 1 Department of Electrical and Electronics Engineering, B V Raju Institute of Technology, Narsapur, India; 2 School of Electronics and Electrical, Lovely Professional University, Phagwara, Punjab, India; 3 Department of Electrical Engineering, I. K. Gujral Punjab Technical University, Main Campus, Kapurthala, Punjab, India; 4 Manipal Institute of Technology, Manipal Academy of Higher Education, Manipal, India; ICFAI Foundation for Higher Education Faculty of Science and Technology, INDIA

## Abstract

The recent development of power electronic devices that utilizes the advantages of wideband gap semiconductor components for electrical conversion is expected to create a new class of reliable and efficient inverters. However, the photovoltaic inverter is considered a critical component in grid-connected PV systems with respect to reliability performance. Therefore, this paper proposes a wide-bandgap semiconductor (WBS) based photovoltaic inverter, specifically utilizing silicon carbide (SiC) MOSFETs and gallium nitride (GaN) HEMTs, to enhance reliability and performance. In this paper, reliability-oriented performance is evaluated on a grid connected 3 kW photovoltaic (PV) inverter system under real-time Mission Profile (MP) in India. Environmental factors are assessed based on a one-minute resolution yearly MP, in which solar irradiance (SI) and ambient temperature (AT) are considered. To model the lifetime with a two-Parameter Weibull distribution, the Monte Carlo simulation (MCS) is utilized to produce 10,000 samples with 5% parameter variation. The B_10_ lifetime a reliability measure indicating the time by which 10% of population are expected to fail, is calculated for the Narsapur, Indian location and analyzed with regard to performance metrics, such as PV power, switch losses, inverter efficiency, and output power. In addition, this study compares the performance of conventional IGBT, super junction MOSFET (SJ-M), silicon carbide (SiC) MOSFET (SiC-M), and gallium nitride (GaN) HEMT (GaN-H) based inverters. Results show that WBG semiconductors can increase reliability and efficiency by eliminating 40% of losses compared to the conventional IGBTs.

## Introduction

The need for sustainable energy sources increased the interest in photovoltaic (PV) systems, with grid connected inverters plays a vital role in energy conversion. The traditional silicon IGBT based inverters are affected by efficiency limitations due to the switching losses. Recent advancements in the WBS technology, including SiC-M and GaN-H are the promising alternatives with their superior thermal and electrical performance. The WBS will significantly impact the reliability performance of the PV inverter. The major types PV inverters are tabulated in Table 1.

**Table 1:** Types of PV Inverters [1].

Category	Inverter Topology
AC-Module Inverter	H-Bridge Type
String Inverter	H-Bridge Type, T-Type, NPC Type
Multistring Inverter	H-Bridge Type, NPC Type
Centralized Inverter	T-Type

The integration of WBS will improve the performance of the inverters. The larger bandgap of WBS enables the operation with lower intrinsic carrier concentration which improves the switching performance even at higher temperatures. This results in higher critical electric field strength about 10 times greater than silicon, thereby it permits the use of thinner drift regions for the same blocking voltage. The superior thermal conductivity of WBS provides the efficient heat dissipation, this leads to the reduction in junction temperature and improves the device lifetime. The work reported by W. M. Hamanah et al., [2] presented an evaluation of various advanced WBS, such as GaN and SiC. These are utilized in a DC drive system for solar power applications. In addition, it demonstrates their implementation in a heliostat unit. The research carried out by W. Van De Sande et al., [3] explores the thermal-mechanical stress generated by the bond wires and die attach wires of certain GaN and SiC MOSFETs in photovoltaic systems. It emphasizes the need for reliable electronic components in urban areas. Through an electro-thermal simulation, it can also analyze junction losses caused by clear and cloudy skies. The investigation undertaken by S. S. Kshatri et al., [4] presents the development of a PV inverter that utilizes a hybrid power module that consists of a SiC/IGBT dual anti-parallel diode and a Si-IGBT hybrid power transistor. This technology can help improve the efficiency of the system and address the financial concerns that come with using SiC components. The findings presented by by A. M. Ganose et al., [5] explores the challenges encountered by wide-band semiconductors when it comes to achieving optoelectronic capabilities that can compete with small-bandgap devices.

The work reported by E. Gurpinar et al., [6] presented the thermal loading of gallium nitride HEMTs and IGBTs in three-level passive PV inverters. In this paper yearly MP of these components, such as solar irradiance and ambient temperature are considered. Finally, comparison between the performance of GaN HEMTs and Si IGBTs with different thermal interface materials are presented. A report on the reliability analysis of 1500 V PV inverters is presented in [7]. The reliability issues are caused by the increasing load stress on the power devices when the maximum DC voltage is extended from 1000 to 1500 V. To address these issues, this paper proposed silicon carbide MOSFETs with variable gate resistance. The approach proposed by D. W. Cunningham et al., [8] evaluated the advantages of utilizing gallium nitride and silicon carbide in power electronic components for photovoltaic systems. It highlights their capability to produce reliable, lightweight, and efficient devices. The work reported by S. A. Ansari et al., [9] compared the cost and efficiency of soft and hard-switched DC-DC boost converters using gallium nitride and SiC devices. The observations made by O. Idbouhouch et al., [10] analyzed the degradation and efficiency of PV inverters in arid regions, which highlights the need to consider their dependability. Inverters with a yearly failure rate of 1–15% are examined. The study utilized a monitoring technique to analyze the efficiency of PV inverters. It compared the actual outputs with the predicted outputs, which revealed that the Sandia model accurately models typical operation of the devices. The comparison of Si with SJ, SiC and GaN in tabulated in Table 2.

**Table 2:** Comparison of Si Vs SJ Vs SiC Vs GaN [11,12].

Material	Bandgap Energy, EG (eV)	Critical Electric Field, E_crit_ (MV/cm)	Electron Mobility, μ_e_ (cm²/V·s)	Saturation Velocity, v_s_ (×10⁷ cm/s)	Thermal Conductivity, κ^th^ (W·cm ⁻ ¹·K ⁻ ¹)
**Si**	1.12	0.3	1440	1.0	1.3
**SJ**	~1.1	~0.6–1.0	~1000–1400	~1.0–1.2	~1.3
**SiC**	3.23	2.5	950	2.0	3.7
**GaN**	3.4	3.3	1400	2.4	2.5

J. He et al., [13] presented the issues encountered by 1500-V PV inverters in terms of their reliability. The increasing load stress and thermal loading are some of the issues that can affect this type of device. This issue can lead to higher system costs. Q. Chai et al., [14] proposed a methodology to improve the performance of PV inverters using power smoothing control. This method can be used in combination with active distribution networks’ volt/var control. It also develops new constraints that can limit the apparent power variation of the device. The study also proposes an optimization strategy that can help minimize power losses.

P. Kut et al., [15] reviewed that the European Union’s rapid growth in renewable energy sources, such as solar power, has been driven by the government’s efforts to reduce greenhouse gas emissions. From 2005 to 2019, the number of PV installations has increased from 2.17 gigawatts to over 130 gigawatts. O. Alavi et al., [16] presented the impact of degradation rare in the PV inverter reliability at various climatic conditions. M. J. Abed et al., [17] presented the performance improvement of reliability indices based on loss of load expectations in renewable generation unit. J. H. Choi et al., [18] a hybrid PWM strategy is implemented for the reliability improvement of NPC PV inverters. A reliability based approach for the preventive maintenance of PV system is presented in [19]. T. Ryu et al., [20] proposed the reliability based DPWM approach for the 5 – level T Type PV inverter for the improved performance.

Despite of extensive studies on WBS for PV inverters, along with significant work on reliability of PV inverter, several critical gaps are remains unaddressed. Prior research addressed the reliability and thermal performance of WBG semiconductor such as SiC and GaN for various PV applications. However the research gaps such as limited real time reliability analysis in Indian climatic conditions, comprehensive lifetime evaluation, comparison across different semiconductors are evident in terms of reliability performance. Hence to address these gaps this paper proposes a wide-bandgap semiconductor-based photovoltaic inverter, specifically utilizing SiC-M and GaN-H, to enhance reliability and performance. The objective of the paper is to improve the reliability of the PV inverter with the WBG semiconductor based switches which offer superior efficiency, thermal performance and lifetime in comparison with the conventional Si based switches. In this paper, reliability-oriented performance is evaluated on a grid connected 3 kW photovoltaic (PV) inverter system under real-time MPs in India. Environmental factors are assessed based on a one-minute resolution yearly MP, in which solar irradiance and ambient temperature are considered. To model the lifetime with a two-Parameter Weibull distribution, the MCS is utilized to produce 10,000 samples with 5% parameter variation. The B_10_ lifetime a reliability measure indicating the time by which 10% of population are expected to fail, is calculated for the Narsapur, Indian location and analyzed with regard to performance metrics, such as PV power, switch losses, inverter efficiency, and output power. In addition, this study compares the performance of conventional IGBT, SJ-M, SiC-M and GaN-H based inverters. Due to the superior characteristics of WBS, i.e., SiC-M and GaN-H, enhances the inverter reliability by reducing switch losses, conduction losses, and thermal stress.

### Reliability oriented performance evaluation methodology

**Fig 1:**
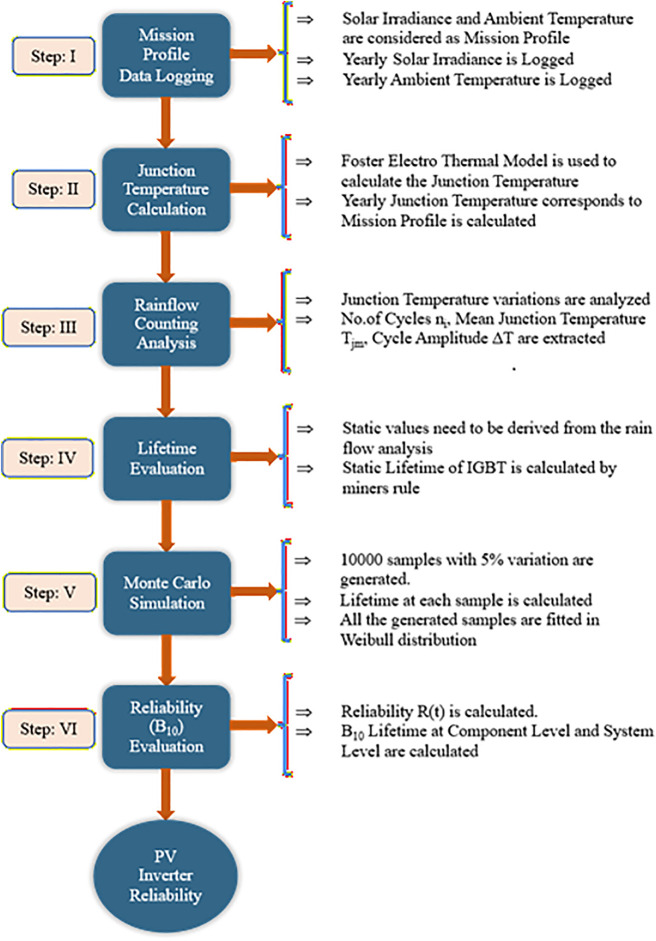
Key procedure steps.

In the PV inverter, the semiconductor switch is considered the most crucial component, making reliability-oriented performance a major concern. Hence in this paper, reliability oriented performance evaluation methodology is presented. Initially, the reliability oriented performance is evaluated at the individual switch level, and then by using a series reliability block diagram approach, the inverter level is evaluated. This approach involves analysing the performance under MP conditions over a period of one year. The key procedure steps are presented in Fig 1.

**Step: 1** – MP Data Logging – One-minute resolution yearly MP, in which SI and AT are considered.

**Step: 2** – Junction Temperature Extraction – The temperature at the junction layers of the semiconductor switches will be extracted using the foster electro thermal model. The mathematical representation for the junction temperature (JT) at any given moment is delineated in Eq. 1 [21].


$$T_{j}=\;Z_{th(j-c)}*\;P_{T}+\;T_{C}$$ (1)

where

- Z_th(j−c)_ = Junction to case Thermal Impedance
- P_T_ = Total Power Loss
- Tc = Case Temperature

The mathematical formulation for the Case Temperature (Tc) is elucidated in Eq. 2.


$$T_{C}=T_{a}+(Z_{th(c-h)}+\;Z_{th(h-a)})*\;P_{T}$$ (2)

Where

- T_a_ = Ambient Temperature
- Z_th(c−h)_ = Case to Heat Sink Thermal Impedance
- Z_th(h−a)_ = Heat sink to Ambient Thermal Impedance

The power dissipation within the semiconductor switch is attributable to thermal energy loss. Predominantly, there exist two categories of power losses, which are

- Conduction Losses (P_c_)
- Switching Losses (P_s_)

The total power loss is determined utilizing the Eq. 3.


$$P_{T}=\;P_{S}+\;P_{C}$$ (3)

The Switching loss is determined utilizing the Eq. 4.


$$P_{S}=\left(E_{on}+\;E_{off}\right)\times f$$ (4)

where

- f = Fundamental switching frequency,
- E_on_ = Turn-on loss
- E_off_ = Turn-off loss.

**Step: 3** – Rainflow Counting Assessment (RCA) –The variations in the extracted JT are caused by the irregular nature of MP. A cycle counting algorithm is required to assess these variations. Hence in this work Rainflow Counting Assessment (RCA) is utilized. From this assessment Total number of cycles, mean temperature and amplitude cycle are evaluated. [22]

**Step: IV** – Lifetime Evaluation – Lifetime is evaluated using miners rule as per Eq. 5


$$Life\;Time\;(LT)=\;\frac{\text{1}}{\sum \frac{{Total\;No.of\;Cycles\;(n}_{i})}{No.of\;Cycle\;to\;failure\;(N_{fi})}\;}$$ (5)

Where

- Total number of cycles are evaluated using RCA.
- No.of Cycles to failure N_f_ is evaluated using bayerers model [23] as per Eq. 6

$$N_{f}=A{\left({\Delta T}_{j}\right)}^{\beta_{\text{1}}}.e^{\frac{\beta_{\text{2}}}{\left(T_{j}+\;\text{2}\text{7}\text{3}K\right)\;}}.\;{t_{on}}^{\beta_{\text{3}}}.\;I^{\beta_{\text{4}}}.\;V^{\beta_{\text{5}}}.\;D^{\beta_{\text{6}}}$$ (6)

**Step: V** – Monte Carlo Simulation – To model the lifetime with a two-Parameter Weibull distribution, the Monte Carlo simulation is utilized to produce 10,000 samples with 5% parameter variation.

**Step: VI** – Reliability (B_10_) Evaluation – The reliability function of the generated samples is calculated by fitting them to the Weibull distribution with 95% confident bounds. [23,24]

The individual switch level reliability is evaluated as per Eq. 7


$$R_{i}(t)=\;e^{{-\left(\frac{t}{\propto }\right)}^{\gamma }}$$ (7)

where ∝ is Scale Parameter (characteristic life, i.e., 63.2% of the population has failed)

γ is Shape Parameter (Failure rate behaviour over time)

The inverter level reliability is evaluated as per Eq. 8


$$R_{total}(t)=\;\prod_{i=\text{1}}^{n}R_{i}(t)$$ (8)

Finally, B_10_ lifetime is evaluated as per Eq. 9.


$$B_{x}=\;{\left[\text{ln}\left(\frac{\text{1}\text{0}\text{0}}{\text{1}\text{0}\text{0}-x}\right)\times (\propto {)}^{\gamma }\right]}^{\frac{\text{1}}{\gamma }}$$ (9)

were

x is percentage of population

∝ is Scale Parameter

γ is Shape Parameter.

### Case system

**Fig 2:**
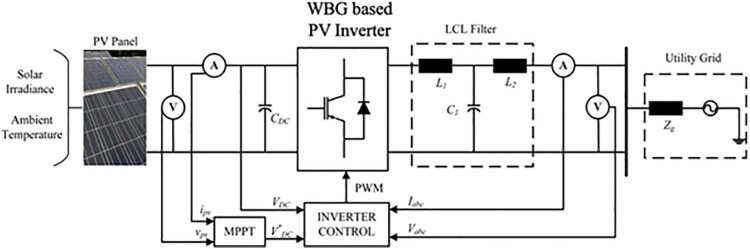
Grid Connected 3-kW Test Case.

This paper proposes a wide-bandgap semiconductor-based photovoltaic inverter, specifically utilizing SiC-M and GaN-H, to enhance reliability and performance. These wideband gap semiconductors exhibits the improved performance over the conventional semiconductor, including efficiency, thermal conductivity etc. utilization of these wideband gap semiconductors are expected to reduce the power loss and improve the reliability of the grid connected inverter system, which is more suitable in variable environmental conditions. The proposed system is implemented on a grid connected 3 kW photovoltaic (PV) inverter system under real-time MPs in India as shown in Fig 2. The details of semiconductor switches and the system parameters of the test case are tabulated in Table 3.

**Table 3:** Details of the Test Case.

S.No	Item	Model & Model	Rating
1.	Si-IGBT	Infineon -IGW30N60H3	600 V, 30 A
2.	SJ – MOSFET	Infineon IPB65R065C7	650 V, 33 A
3.	SiC – MOSFET	Infineon - IMBG65R163M1H	650 V, 31 A
4.	GaN – HEMT	Infineon – GS66508B-TR	650 V, 30 A
5.	Solar Panel	Ameresco - BP365	60 W(P_max_)
6.	System Voltage	--	230 V
7.	System Frequency	--	50 Hz
8.	System DC Capacitance	--	1.5 x 10^−3^ F

**Fig 3:**
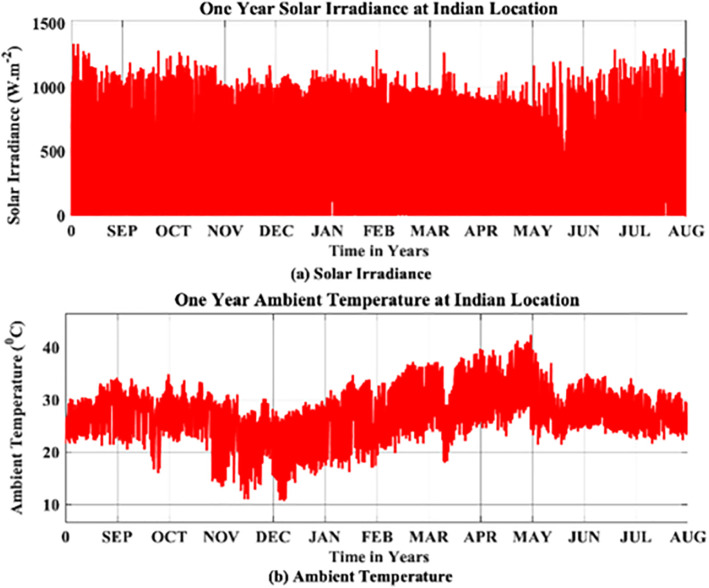
Environmental Factors (Mission Profile).

The environmental factors based on a one-minute resolution yearly MP, in which SI and AI are meticulously recorded from 1^st^ September 2023–31^st^ August 2024 at Narsapur, Medak District, Telangana, India as shown in Fig 3. The yearly MP logging process will ensures the accuracy and captures the variation trends of environment which will impact the systems performance.

## Results and discussions

In this paper, reliability-oriented performance is evaluated on a grid connected 3 kW photovoltaic (PV) inverter system under real-time MPs in Narsapur, India. To model the lifetime with a two-Parameter Weibull distribution, the MCS is utilized to produce 10,000 samples with 5% parameter variation. The B_10_ lifetime a reliability measure indicating the time by which 10% of population are expected to fail, is calculated for the Narsapur, Indian location and analyzed with regard to performance metrics, such as PV power, switch losses, inverter efficiency, and output power. The reliability-oriented performance is evaluated under the following cases

- Performance Evaluation with Si-IGBT based PV Inverter
- Performance Evaluation with Sj-MOSFET based PV Inverter
- Performance Evaluation with SiC-MOSFET based PV Inverter
- Performance Evaluation with GaN-HEMT based PV Inverter

### Performance evaluation with Si-IGBT based PV inverter

**Fig 4:**
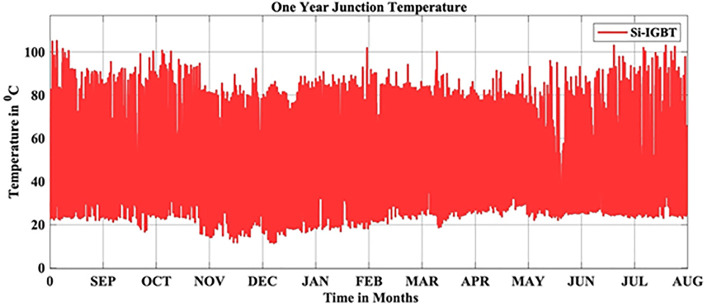
Si-IGBT Based PV Inverter JT.

In this scenario, the reliability-oriented performance of grid connected 3 kW photovoltaic (PV) inverter system considering Si-IGBT based PV Inverter is evaluated. The JT corresponds to the real-time MPs is extracted using the FET model as depicted in Fig 4. Over the span of a year, the average JT recorded over a year is 56.04 °C.

**Fig 5:**
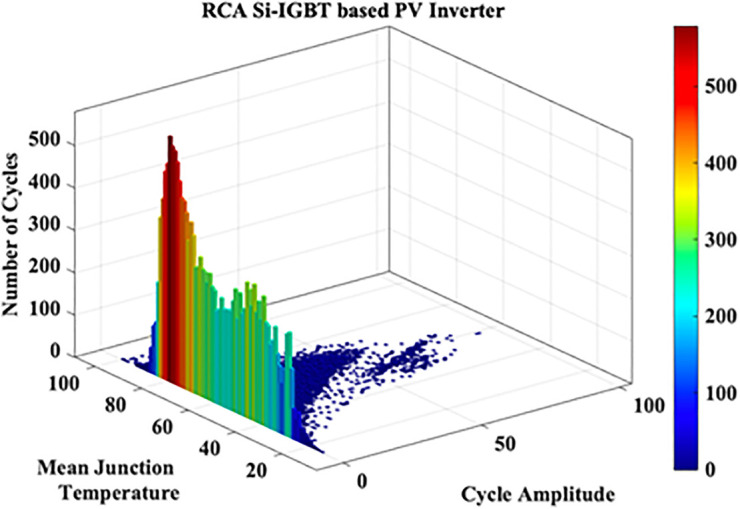
RCA Si-IGBT based PV Inverter.

These variations in the extracted JT arise from the irregular nature of MP. A cycle counting algorithm is required to assess these variations. Hence in this work Rainflow Counting Assessment (RCA) is utilized. From this assessment, RCA parameters such as total number of cycles, mean temperature and amplitude cycle are evaluated as shown in Fig 5. The RCA parameters are tabulated in Table 4.

**Table 4:** Static B_10_ Lifetime Si-IGBT based PV Inverter.

S.No	RCA Parameter	Obtained Value
1	Total Number of Cycles	34981
2	Mean Temperature	60.07 °C
3	Amplitude Cycle	6.08 °C

**Fig 6:**
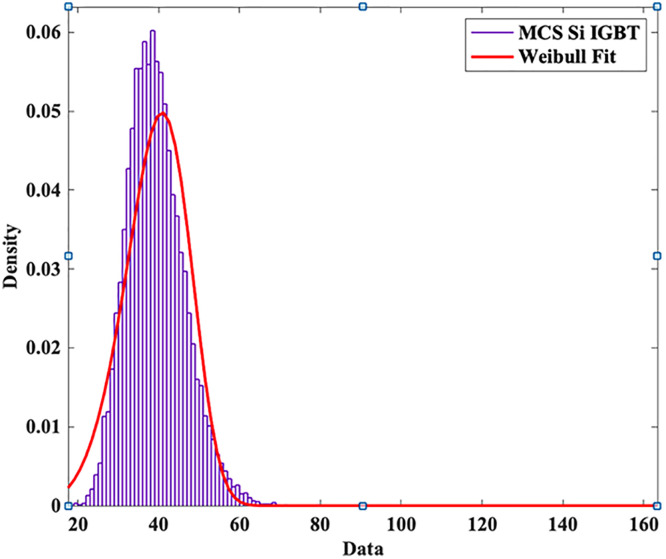
MCS Si-IGBT based PV Inverter.

To model the lifetime with a two-Parameter Weibull distribution, the Monte Carlo simulation is utilized to produce 10,000 samples with 5% parameter variation and life time is calculated using the Eq. 5 and Eq. 6 as shown in Fig 6.

**Fig 7:**
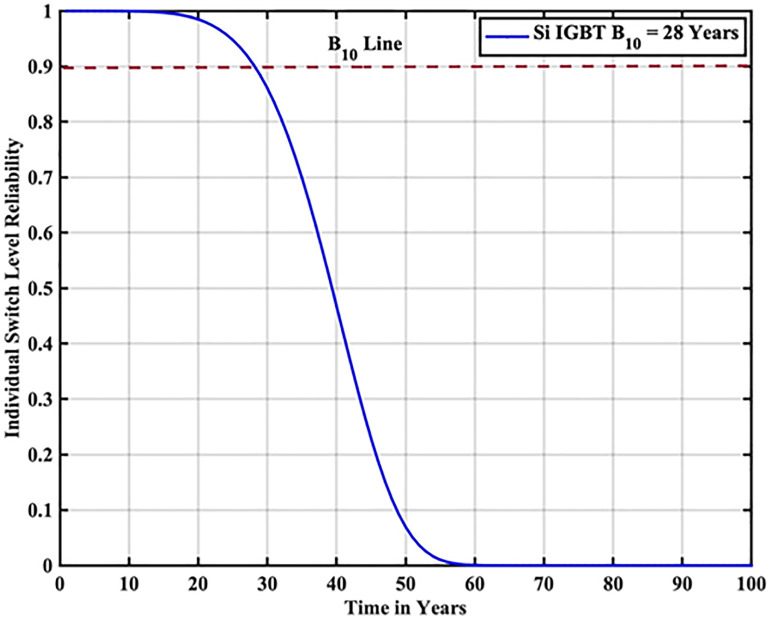
Individual Switch Level Reliability Si-IGBT based PV Inverter.

**Fig 8:**
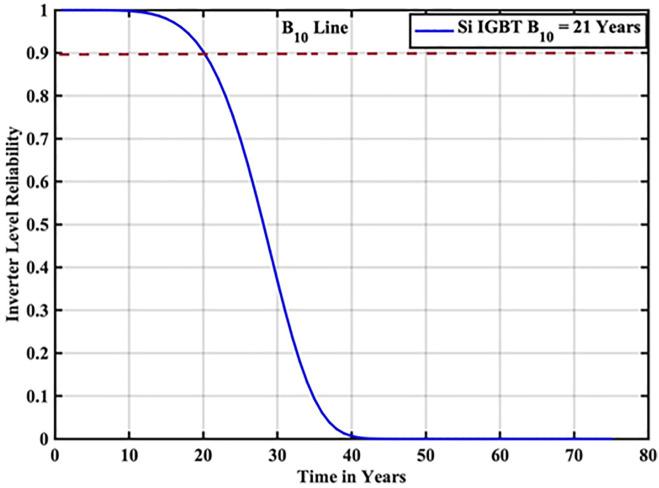
Inverter Level Reliability Si-IGBT based PV Inverter.

The reliability function of the generated samples is calculated by fitting them to the Weibull distribution. The individual switch level reliability is evaluated as per Eq. 7, The inverter level reliability is evaluated as per Eq. 8 as depicted in Figs 7 and 8 respectively.

**Fig 9:**
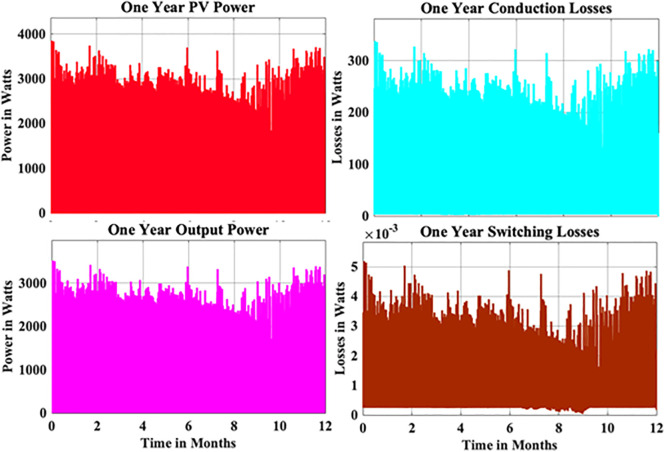
Yearly Performance Metrics Si-IGBT Based PV Inverter.

From the above reliability curves it is observed that the B_10_ lifetime at individual switch level is 28 year, while the B_10_ lifetime at inverter level is 21 years. In addition to reliability assessment, performance metrics such as such as PV power, conduction losses, switching losses, total switch losses, output power and inverter efficiency are evaluated as shown in Fig 9. The yearly average PV power, conduction losses, switching losses, total switch losses, output power and inverter efficiency are tabulated in Table 5. These metrics are significant in understanding the overall performance of Si-IGBT based PV inverter.

**Table 5:** Yearly Average Parameters Si-IGBT based PV Inverter.

S.No	Parameter	Value
1	PV Power	1623.96 W
2	Conduction Losses	118.73 W
3	Switching Losses	0.00148 W
4	Total Switch Losses	118.73 W
5	Output Power	1505.23 W
6	Efficiency	92.69%

### Performance evaluation with SJ-MOSFET based PV inverter

**Fig 10:**
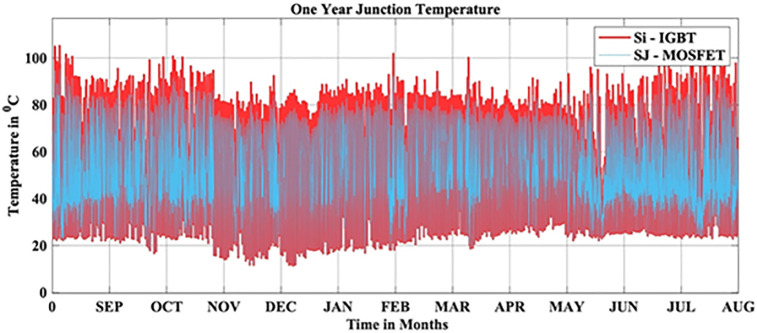
SJ-MOSFET based PV Inverter JT.

In this scenario, the reliability-oriented performance of grid connected 3 kW photovoltaic (PV) inverter system considering SJ-MOSFET based PV Inverter is evaluated. The JT corresponds to the real-time MPs is extracted using the FET model as depicted in Fig 10. Over the span of a year, the average JT recorded over a year is 53.10 °C.

**Fig 11:**
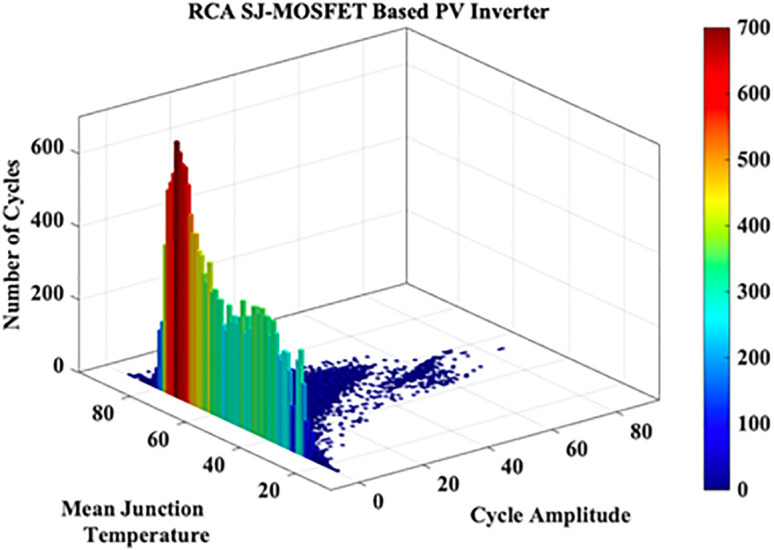
RCA SJ-MOSFET based PV Inverter.

These variations in the extracted JT arise from the irregular nature of MP. A cycle counting algorithm is required to assess these variations. Hence in this work Rainflow Counting Assessment (RCA) is utilized. From this assessment, RCA parameters such as total number of cycles, mean temperature and amplitude cycle are evaluated as shown in Fig 11. The RCA parameters are tabulated in Table 6.

**Table 6:** Static B_10_ Lifetime SJ-MOSFET based PV Inverter.

S.No	RCA Parameter	Obtained Value
1	Total Number of Cycles	35248
2	Mean Temperature	56.60 °C
3	Amplitude Cycle	5.78 °C

**Fig 12:**
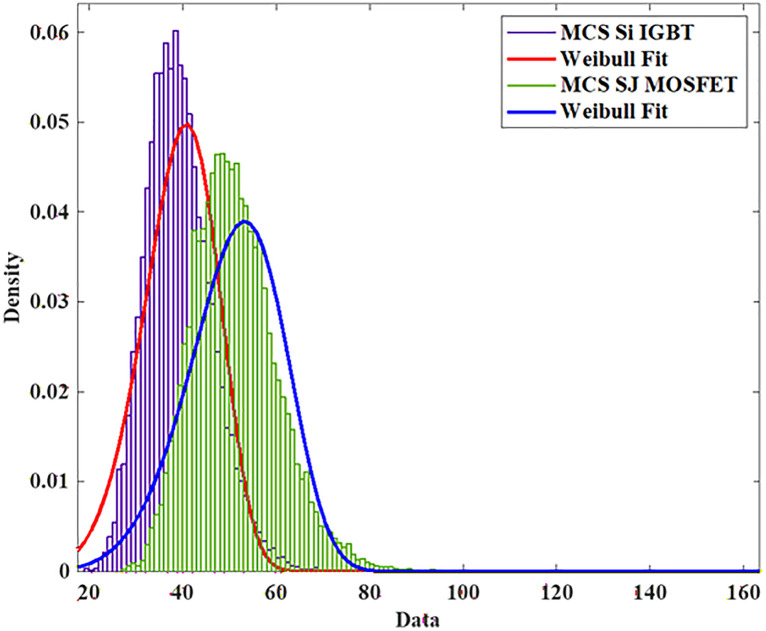
MCS SJ-MOSFET based PV Inverter.

To model the lifetime with a two-Parameter Weibull distribution, the Monte Carlo simulation is utilized to produce 10,000 samples with 5% parameter variation and life time is calculated using the Eq. 5 and Eq. 6 as shown in Fig 12.

**Fig 13:**
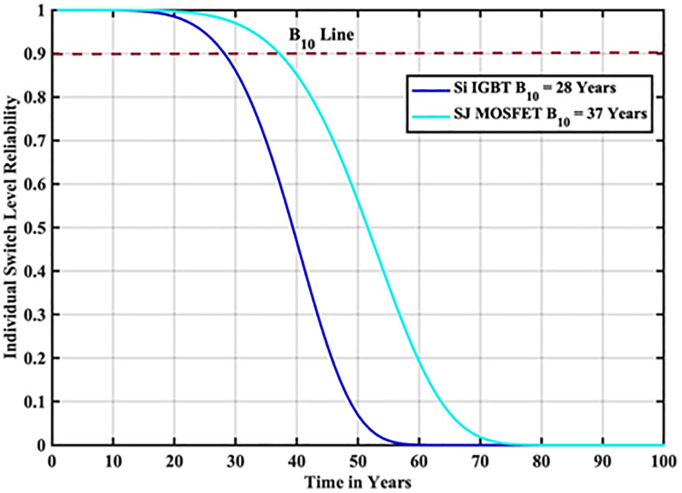
Individual Switch Level Reliability SJ-MOSFET based PV Inverter.

**Fig 14:**
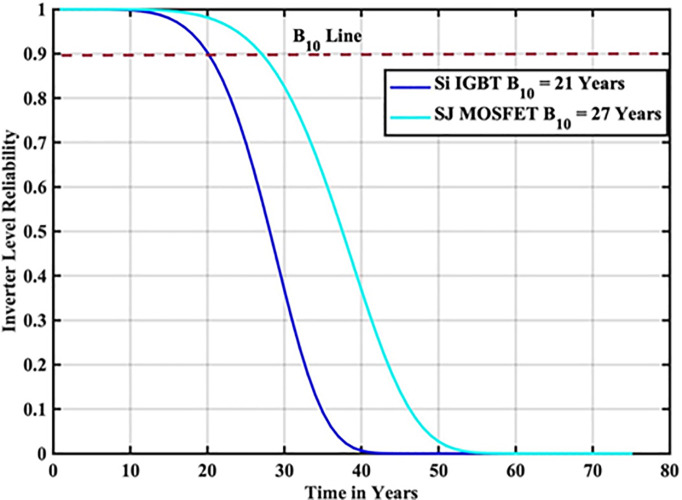
Inverter Level Reliability SJ-MOSFET based PV Inverter.

The reliability function of the generated samples is calculated by fitting them to the Weibull distribution. The individual switch level reliability is evaluated as per Eq. 7, The inverter level reliability is evaluated as per Eq. 8 as depicted in Figs 13 and 14 respectively.

**Fig 15:**
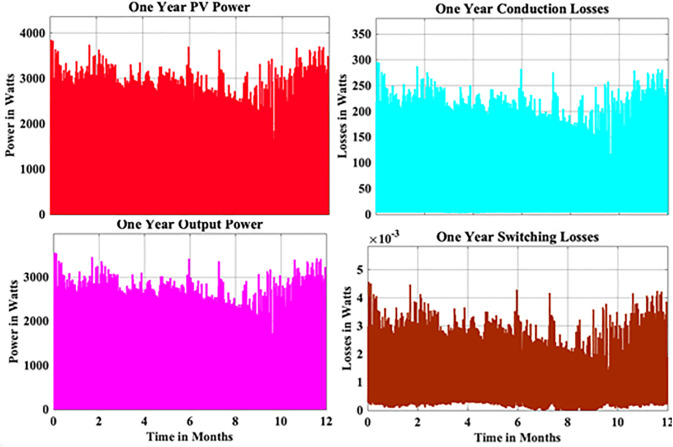
Yearly Performance Metrics SJ-MOSFET based PV Inverter.

From the above reliability curves it is observed that the B_10_ lifetime at individual switch level is 37 year, while the B_10_ lifetime at inverter level is 27 years. In addition to reliability assessment, performance metrics such as such as PV power, conduction losses, switching losses, total switch losses, output power and inverter efficiency are evaluated as shown in Fig 15. The yearly average PV power, conduction losses, switching losses, total switch losses, output power and inverter efficiency are tabulated in Table 7. These metrics are significant in understanding the overall performance of Si-IGBT based PV inverter.

**Table 7:** Yearly Average Parameters SJ-MOSFET based PV Inverter.

S.No	Parameter	Value
1	PV Power	1623.96 W
2	Conduction Losses	106.12 W
3	Switching Losses	0.00133 W
4	Total Switch Losses	106.12 W
5	Output Power	1517.83 W
6	Efficiency	93.47%

In this scenario, the reliability-oriented performance of grid connected 3 kW photovoltaic (PV) inverter system considering SJ-MOSFET based PV Inverter exhibited the improved performance than the conventional Si-IGBT based PV Inverter. The JT is decreased from 56.04 °C to 53.10 °C. The B_10_ lifetime at individual switch level is improved from 28 Years to 37 year, while the B_10_ lifetime at inverter level is improved from 21 Years to 27 years. In addition to reliability assessment, performance metrics such as such as PV power, conduction losses, switching losses, total switch losses, output power are also improved. Inverter efficiency is improved from 92.69% to 93.47%.

### Performance evaluation with SiC-MOSFET based PV inverter

**Fig 16:**
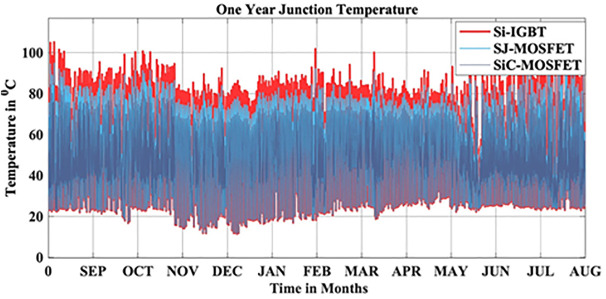
SiC-MOSFET based PV Inverter JT.

In this scenario, the reliability-oriented performance of grid connected 3 kW photovoltaic (PV) inverter system considering SiC-MOSFET based PV Inverter is evaluated. The JT corresponds to the real-time MPs is extracted using the FET model as depicted in Fig 16. Over the span of a year, the average JT recorded over a year is 50.15 °C.

**Fig 17:**
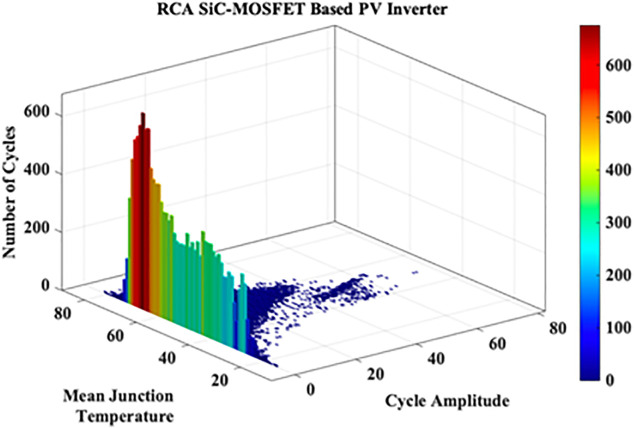
RCA SiC-MOSFET based PV Inverter.

These variations in the extracted JT arise from the irregular nature of MP. A cycle counting algorithm is required to assess these variations. Hence in this work Rainflow Counting Assessment (RCA) is utilized. From this assessment, RCA parameters such as total number of cycles, mean temperature and amplitude cycle are evaluated as shown in Fig 17. The RCA parameters are tabulated in Table 8.

**Table 8:** Static B_10_ Lifetime SiC-MOSFET based PV Inverter.

S.No	RCA Parameter	Obtained Value
1	Total Number of Cycles	35562
2	Mean Temperature	53.12 °C
3	Amplitude Cycle	5.35 °C

**Fig 18:**
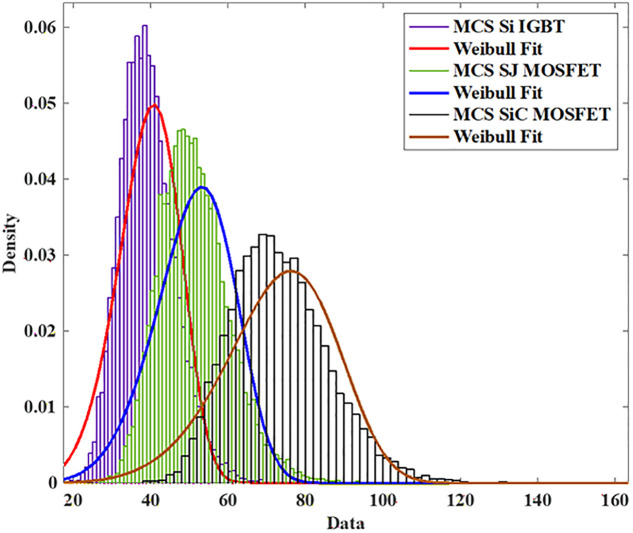
MCS SiC-MOSFET based PV Inverter.

To model the lifetime with a two-Parameter Weibull distribution, the Monte Carlo simulation is utilized to produce 10,000 samples with 5% parameter variation and life time is calculated using the Eq. 5 and Eq. 6 as shown in Fig 18.

**Fig 19:**
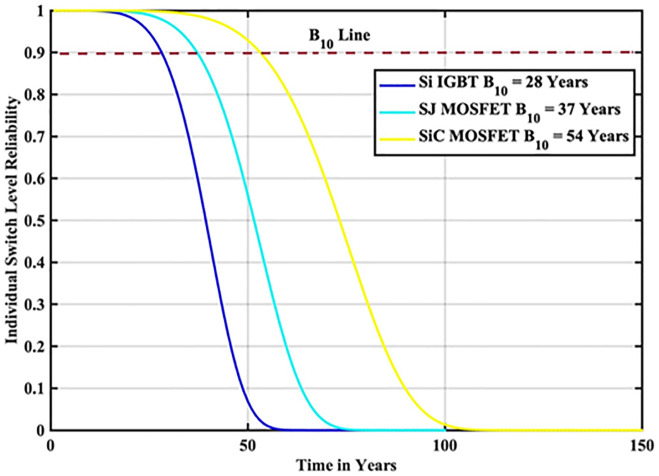
Individual Switch Level Reliability SiC-MOSFET based PV Inverter.

**Fig 20:**
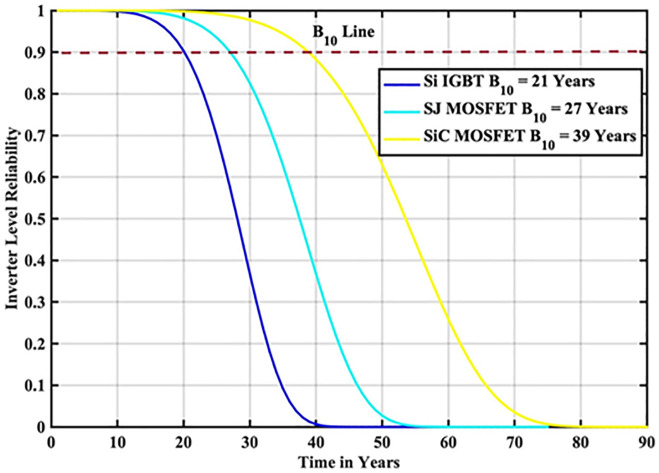
Inverter Level Reliability SiC-MOSFET based PV Inverter.

The reliability function of the generated samples is calculated by fitting them to the Weibull distribution. The individual switch level reliability is evaluated as per Eq. 7, The inverter level reliability is evaluated as per Eq. 8 as depicted in Figs 19 and 20 respectively.

**Fig 21:**
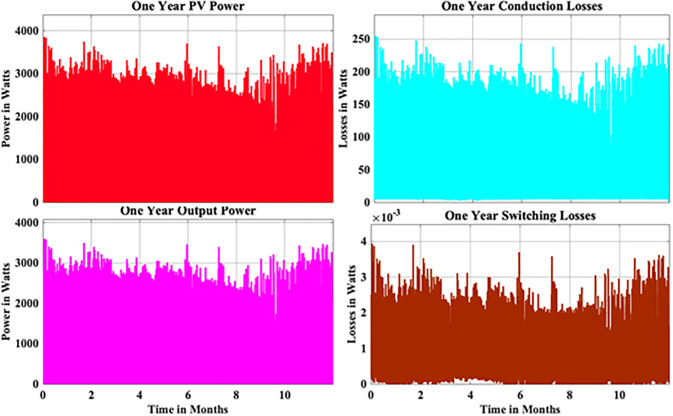
Yearly Performance Metrics SiC-MOSFET based PV Inverter.

From the above reliability curves it is observed that the B_10_ lifetime at individual switch level is 54 year, while the B_10_ lifetime at inverter level is 39 years. In addition to reliability assessment, performance metrics such as such as PV power, conduction losses, switching losses, total switch losses, output power and inverter efficiency are evaluated as shown in Fig 21. The yearly average PV power, conduction losses, switching losses, total switch losses, output power and inverter efficiency are tabulated in Table 9. These metrics are significant in understanding the overall performance of Si-IGBT based PV inverter.

**Table 9:** Yearly Average Parameters SiC-MOSFET based PV Inverter.

S.No	Parameter	Value
1	PV Power	1623.96 W
2	Conduction Losses	93.51 W
3	Switching Losses	0.00117 W
4	Total Switch Losses	93.51 W
5	Output Power	1530.44 W
6	Efficiency	94.24%

In this scenario, the reliability-oriented performance of grid connected 3 kW photovoltaic (PV) inverter system considering SiC-MOSFET based PV Inverter exhibited the improved performance than the conventional Si-IGBT based PV Inverter. The JT is decreased from 56.04 °C to 50.15 °C. The B_10_ lifetime at individual switch level is improved from 28 Years to 54 year, while the B_10_ lifetime at inverter level is improved from 21 Years to 39 years. In addition to reliability assessment, performance metrics such as such as PV power, conduction losses, switching losses, total switch losses, output power are also improved. Inverter efficiency is improved from 92.69% to 94.24%.

### Performance evaluation with GaN-HEMT based PV inverter

**Fig 22:**
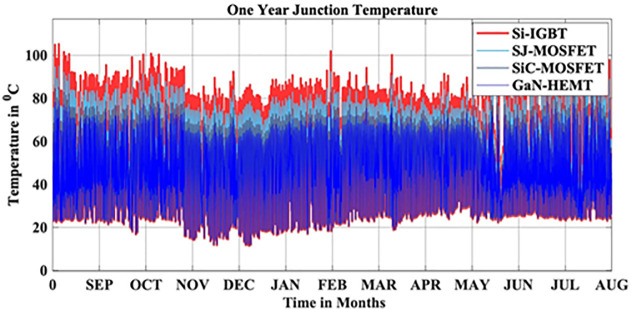
GaN-HEMT based PV Inverter JT.

In this scenario, the reliability-oriented performance of grid connected 3 kW photovoltaic (PV) inverter system considering GaN-HEMT based PV Inverter is evaluated. The JT corresponds to the real-time MPs is extracted using the FET model as depicted in Fig 22. Over the span of a year, the average JT recorded over a year is 48.31 °C.

**Fig 23:**
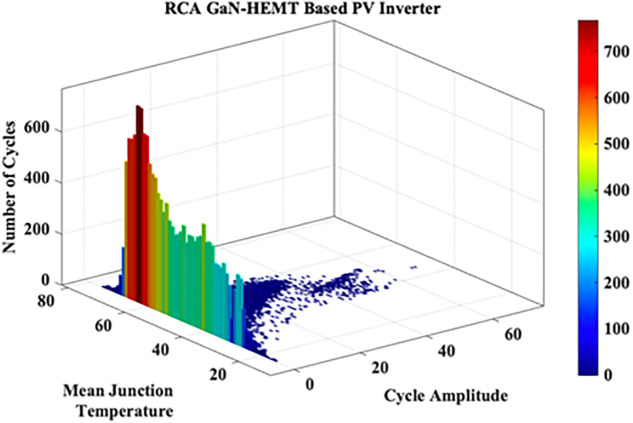
RCA GaN-HEMT based PV Inverter.

These variations in the extracted JT arise from the irregular nature of MP. A cycle counting algorithm is required to assess these variations. Hence in this work Rainflow Counting Assessment (RCA) is utilized. From this assessment, RCA parameters such as total number of cycles, mean temperature and amplitude cycle are evaluated as shown in Fig 23. The RCA parameters are tabulated in Table 10.

**Table 10:** Static B_10_ Lifetime GaN-HEMT based PV Inverter.

S.No	RCA Parameter	Obtained Value
1	Total Number of Cycles	35810
2	Mean Temperature	50.97 °C
3	Amplitude Cycle	5.22 °C

**Fig 24:**
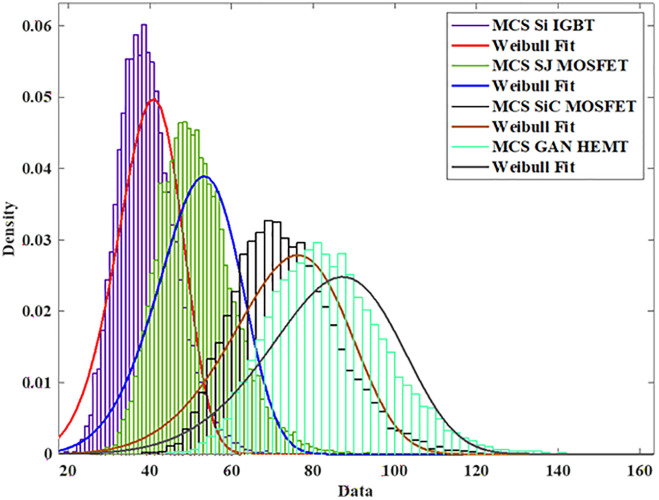
MCS GaN-HEMT based PV Inverter.

To model the lifetime with a two-Parameter Weibull distribution, the Monte Carlo simulation is utilized to produce 10,000 samples with 5% parameter variation and life time is calculated using the Eq. 5 and Eq. 6 as shown in Fig 24.

**Fig 25:**
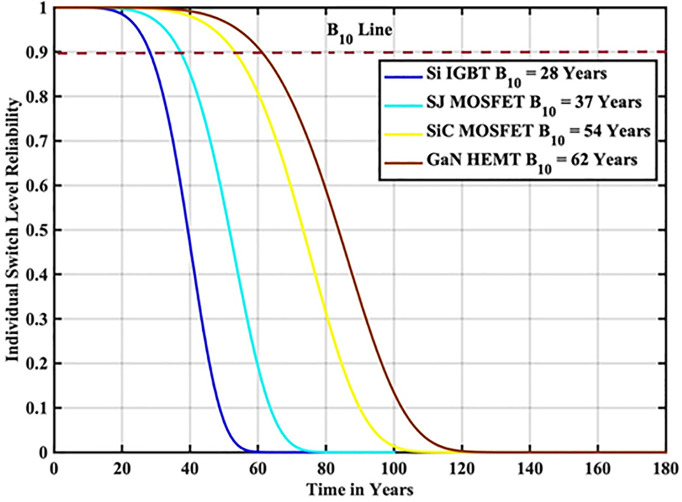
Individual Switch Level Reliability GaN-HEMT based PV Inverter.

**Fig 26:**
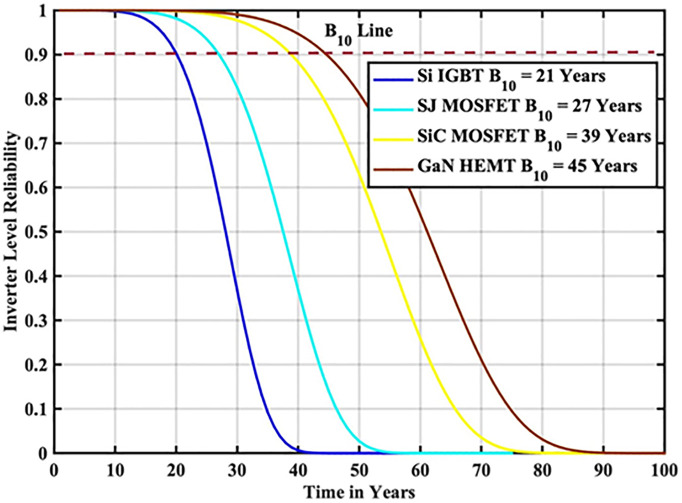
Inverter Level Reliability GaN-HEMT based PV Inverter.

The reliability function of the generated samples is calculated by fitting them to the Weibull distribution. The individual switch level reliability is evaluated as per Eq. 7, The inverter level reliability is evaluated as per Eq. 8 as depicted in Figs 25 and 26 respectively.

**Fig 27:**
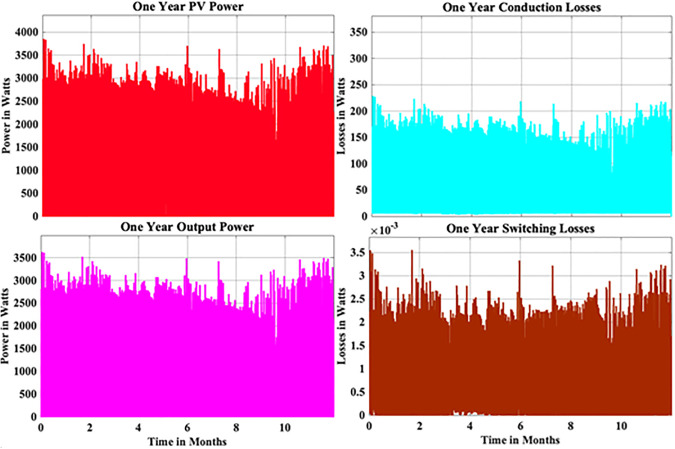
Yearly Performance Metrics GaN-HEMT based PV Inverter.

From the above reliability curves it is observed that the B_10_ lifetime at individual switch level is 62 year, while the B_10_ lifetime at inverter level is 45 years. In addition to reliability assessment, performance metrics such as such as PV power, conduction losses, switching losses, total switch losses, output power and inverter efficiency are evaluated as shown in Fig 27. The yearly average PV power, conduction losses, switching losses, total switch losses, output power and inverter efficiency are tabulated in Table 11. These metrics are significant in understanding the overall performance of Si-IGBT based PV inverter.

**Table 11:** Yearly Average Parameters GaN-HEMT based PV Inverter.

S.No	Parameter	Value
1	PV Power	1623.96 W
2	Conduction Losses	85.63 W
3	Switching Losses	0.00107 W
4	Total Switch Losses	85.63 W
5	Output Power	1538.32 W
6	Efficiency	94.73%

In this scenario, the reliability-oriented performance of grid connected 3 kW photovoltaic (PV) inverter system considering GaN-HEMT based PV Inverter exhibited the improved performance than the conventional Si-IGBT based PV Inverter. The JT is decreased from 56.04 °C to 48.31 °C. The B_10_ lifetime at individual switch level is improved from 28 Years to 62 year, while the B_10_ lifetime at inverter level is improved from 21 Years to 45 years. In addition to reliability assessment, performance metrics such as such as PV power, conduction losses, switching losses, total switch losses, output power are also improved. Inverter efficiency is improved from 92.69% to 94.73%. The reliability of PV Inverter at Indian location for Si-IGBT is reported in literature, a comparison table it presented in Table 12 for validation

**Table 12:** B_10_ Lifetime Comparison at Indian Location.

S.No	Reference	B_10_ Lifetime Component Level	B_10_ Lifetime System Level
1	[25]	30 Years	13 Years
2	[26]	34 Years	18 Years
3	Proposed Si-IGBT	28 Years	21 Years
4	Proposed SJ-MOSFET	37 Years	27 Years
5	Proposed SiC – MOSFET	54 Years	39 Years
6	Proposed GaN-HEMT	62 Years	45 Years

### The practical design implications are listed below

- Increasing the heatsink size improves thermal performance and thereby enhances reliability.
- Oversizing the PV panel increases thermal stress, leading to a reduction in reliability.
- Derating of semiconductor switches reduces thermal stress and thereby improves reliability.

## Conclusion

In this paper, reliability-oriented performance is evaluated on a grid connected 3 kW photovoltaic (PV) inverter system under real-time MPs in Narsapur, India. Environmental factors are assessed based on a one-minute resolution yearly MP, in which solar irradiance and ambient temperature are considered. To model the lifetime with a two-Parameter Weibull distribution, the Monte Carlo simulation is utilized to produce 10,000 samples with 5% parameter variation. The B_10_ lifetime a reliability measure indicating the time by which 10% of population are expected to fail, is calculated for the Narsapur, Indian location and analyzed with regard to performance metrics, such as PV power, switch losses, inverter efficiency, and output power. The proposed wideband gap semiconductor based PV inverter exhibited the improved performance than the conventional Si-IGBT and SJ-MOSFET based PV Inverter. The JT is decreased from 56.04 °C to 48.31 °C. The B_10_ lifetime at individual switch level is improved from 28 Years to 62 year, while the B_10_ lifetime at inverter level is improved from 21 Years to 45 years. In addition to reliability assessment, performance metrics such as such as PV power, conduction losses, switching losses, total switch losses, output power are also improved. Inverter efficiency is improved from 92.69% to 94.73%. The wideband gap semiconductors, SiC-M and GaN-H exhibited the same performance. Due to the scope limitations, the current work addresses only a single location; however, in future work, we plan to extend this analysis to additional sites and provide the comparison and impact of climatic zone on inverter reliability.
